# Foot and lower leg pain in children and adults with cerebral palsy: a population-based register study on 5,122 individuals

**DOI:** 10.1186/s12891-024-07486-y

**Published:** 2024-05-18

**Authors:** Ebba Jarlman, Gunnar Hägglund, Ann I. Alriksson-Schmidt

**Affiliations:** 1 Department of Clinical Sciences, Orthopedics, Lund University, Lund, Sweden; 2 Department of Orthopedics, Helsingborg Hospital, Helsingborg, Sweden

**Keywords:** Cerebral palsy, Pain, Pain intensity, Lower extremities

## Abstract

**Background:**

Pain is common in individuals with cerebral palsy (CP) and the most reported pain site is the foot/lower leg. We analyzed the prevalence of pain in the foot/lower leg and the associations with age, sex, gross motor function, and clinical findings in individuals with CP.

**Method:**

This was a cross-sectional register-study, based on data reported to the Swedish Cerebral Palsy Follow-up Program (CPUP). All participants in CPUP, four years-of-age or older, were included. Pearson chi-square tests and logistic regression were used to analyze the prevalence and degree of pain in the foot/lower leg.

**Results:**

In total, 5,122 individuals were included from the CPUP database: 58% were males and 66% were under 18 years-of-age. Overall, 1,077 (21%) reported pain in the foot/lower leg. The odds ratios (ORs) of pain were higher in females (OR 1.31, 95% confidence interval (CI) 1.13–1.53), individuals who could ambulate (Gross Motor Function Classification System Level I (OR 1.84, CI 1.32–2.57) and II (OR 2.01, CI 1.46–2.79) compared to level V), and in individuals with decreased range of motion of the ankle (dorsiflexion 1–10 degrees (OR 1.43, CI 1.13–1.83) and ≤ 0 degrees (OR 1.46, CI 1.10–1.93) compared to ≥ 20 degrees). With increasing age the OR of pain increased (OR 1.02, CI 1.01–1.03) as well as the reported pain intensity (*p* < 0.001).

**Conclusions:**

Pain in the foot and lower leg appears to be a significant problem in individuals with CP, particularly in those who walk. As with pain in general in this population, both pain intensity and frequency increase with age. The odds of pain in the foot and lower leg were increased in individuals with limited dorsiflexion of the ankle. Given the cross-sectional design causality cannot be inferred and it is unknown if pain causes decreased range of motion of the ankle or if decreased range of motion causes pain. Further research is needed on causal pathways and importantly on prevention.

## Background

Cerebral palsy (CP) is caused by a non-progressive injury to the brain but is associated with progressive musculoskeletal complications. Pain is consistently reported as one of the more frequent secondary conditions [1–7]. The Swedish Cerebral Palsy Follow-up Program (CPUP) is a combined national follow-up program and register that includes regular and systematic assessments to identify early signs of deterioration. CPUP has successfully reduced the prevalence of hip dislocations and severe contractures [8, 9]. However, the effect on pain is unclear. Previous research has indicated that the number of individuals with CP reporting pain ranges from 31 to 77% [1, 10–14].

Pain has been associated with increasing age as well as with female sex, both in studies of children and adults with CP [3, 5, 6, 10, 15–17]. Previous studies based on data from the CPUP have shown that pain in the last four weeks was reported in 32–43% of children and 67% of adults [3, 16, 17]. In addition to frequency of pain, the number of pain sites increased with age: approximately one in three children reported pain in more than one site, compared to three in four adults [16, 17]. Those in pain reported that pain affected their Activities of Daily Living (ADL) or work in 60% of cases, and their sleep in 36–47% [16, 17]. Recurrent musculoskeletal pain has been associated with lower levels of participation in life situations, lower self-reported quality of life, as well as a decline in gross motor function [3, 4, 6, 14, 18–23].

One of the most common pain sites in individuals with CP is the feet and lower legs (from hereon after referred to as foot/lower leg) constituting 36–60% of reported pain sites [3, 16, 17, 20, 24, 25]. However, reported pain site/s is strongly associated with gross motor function classification system (GMFCS) levels [1, 3, 14, 22, 26] such that children with walking ability (i.e., GMFCS levels I-III) report pain in the foot/lower leg to a greater extent than children using wheelchairs (GMFCS levels IV-V) [3, 16, 24]. Spasticity has been described as a common cause of musculoskeletal pain, and the foot/lower leg/knee has been reported as the most common site for spasticity-related pain [7, 13, 14, 27]. Research has also indicated that spasticity of the gastrocsoleus complex is related to the development of muscle contractures and decreased range of motion (ROM) of the ankle [28].

Although pain in CP regardless of pain site is well described, it has been shown that the pain distribution differs by e.g., level of motor function. The mapping of pain by specific body sites is not well understood. To that end, we were interested in studying more specific factors that might be correlated to the painful foot/lower leg. Our aim was to analyze pain in the foot/lower leg in individuals with CP related to age, sex, GMFCS-level, ROM of the ankle, and level of spasticity in the plantar flexors. We also investigated the prevalence of spasticity-reducing treatments and below-the-knee surgery in those who reported pain in the foot/lower leg compared to those who did not.

## Methods

This was a cross-sectional, population-based, CPUP register-study based on data reported in 2018–2019. The CPUP assessment schedule is based on age and GMFCS-level. Children are offered to participate in the CPUP follow-up program as soon as there is suspicion that they might have CP. The actual diagnoses of CP are made at approximately 4 years of age by pediatric neurologists according to the criteria developed by the Surveillance of Cerebral Palsy Network in Europe [26]. Children not fulfilling the inclusion criteria for the diagnosis of CP are then excluded. Both children and adults with CP are eligible to participate in CPUP. More than 95% of children 18 years old or younger with CP in Sweden are followed in CPUP, however, the participation rate for adults is lower due to approximately 15% of individuals with CP choosing to discontinue the program after their 18th birthday.

As part of the CPUP assessments, physical therapists screen for pain. If pain is present, the intensity in the last four weeks in 15 separate body sites is recorded. Pain intensity is graded on an ordinal scale from 0 (no pain) to 5 (very severe pain) for each site. For the purpose of this study, data on pain in the foot/lower leg and total number of painful sites were included in addition to two items on pain interference with ADL and sleep. If the individuals were not able to communicate, the parents or legal guardians served as proxies.

The ROM of the ankle was measured with 90-degree flexion of the hip and knee, using a goniometer. When performing this measurement, the stationary arm of the goniometer was parallel to the anterior margin of the tibia, and the movable arm was parallel to the lateral margin of the foot. The neutral position of the ankle was set as 0 degrees of dorsiflexion and the amount of dorsiflexion measured was registered at the closest 5-degree mark. If the ankle could not come up to neutral position this was reported as a negative degree of dorsiflexion (i.e., -5 degrees). Degree of spasticity in the plantar flexors is classified according to the Modified Ashworth Scale (MAS) from 0 to 4. We dichotomized spasticity into ‘low’ (MAS 0–1) or ‘high’ (MAS ≥ 2). Because pain laterality is not recorded, the decision was made to use the lowest ROM and the highest degree of spasticity recorded. Eleven participants had reported “no pain” and five had left the item on ‘pain present’ blank, yet still recorded pain site/s. In those cases, pain was recoded as ‘pain present’.

Data were collected from the last CPUP assessment performed in 2018–2019 for all participants included. Nine individuals who had undergone below-the-knee surgery within six weeks prior to the assessment were excluded.

The study was approved by the Ethics Board at Lund University (LU 443 − 99, revised 2020).

### Statistics

Descriptive analyses were performed using medians and interquartile ranges (IQR) for continuous variables and raw numbers and percentages for categorical and ordinal variables. “Pain in foot/lower leg’’ was coded as a binary variable (pain/no pain) and categorized as four levels: no pain, mild pain (very mild-mild pain combined), moderate pain, and severe pain (severe-very severe pain combined). Crosstabulations and Pearson chi-square tests were performed. Multicollinearity was assessed with Pearson correlation and Variance Inflation Factor.

Logistic regression analyses were used to estimate unadjusted odds ratios (ORs) of age, sex, GMFCS-level, source of report (self or proxy report), CP-subtype, ROM of the ankle, and spasticity in plantar flexors on pain in the foot/lower leg. Next, a stepwise multiple logistic regression was performed to produce adjusted ORs. Multinominal logistic regression analyses were used to estimate relative risk ratios (RRRs) of the same variables on pain intensity. Listwise deletion was used in the multiple logistic regression analyses. Bonferroni correction was used to adjust the level of significance and defined as *p* < 0.0014 and confidence intervals at 95%. Analyses were performed using IBM SPSS Statistics version 28 and Stata/SE (v 15.1; StataCorp LLC).

## Results

In total, 5,122 individuals were included of which 58% were males. The median age was 14 years (range 4–78, IQR 9–21 years) and 3,370 (66%) were younger than 18 years of age. Baseline characteristics are presented in Table 1.

**Table 1:** Baseline characteristics of the total study population (N = 5122).

Total study population	
**Age intervals, n (%)**	
4–7 yrs	935 (18.3)
8–12 yrs	1299 (25.4)
13–19 yrs	1429 (27.9)
20–35 yrs	1063 (20.8)
36–50 yrs	277 (5.4)
> 50 yrs	119 (2.3)
**Sex**	
Male	2953 (57.7)
Female	2169 (42.3)
GMFCS*	
I	1869 (36.5)
II	929 (18.1)
III	548 (10.7)
IV	819 (16.0)
V	957 (18.7)
**Subtype of cerebral palsy**	
Spastic	3921 (76.6)
Dyskinetic	605 (11.8)
Ataxic	221 (4.3)
Mixed/non-classifiable	282 (5.5)
Missing data	93 (1.8)
**Source of report**	
Self-report	2797 (54.6)
Proxy-report	2181 (42.6)
Missing data	144 (2.8)

*GMFCS: Gross motor function classification system

### Pain

Pain in one or more of the 15 potential pain sites was reported in 2,693 (53%) participants, 2,288 (45%) reported no pain, and 141 (3%) had missing pain data. Pain increased from 36% in 4–7-year-olds to 76% in individuals > 50 years of age. In total, pain was reported in 69% of adults compared to 44% in those under the age of 18 years. Regardless of pain site/s, 57% of individuals in pain reported that pain affected their ADL and 39% that pain affected their sleep.

### Pain in the foot/lower leg

Pain in the foot/lower leg was reported in 1,077 individuals, which corresponds to 21% of the total study population and 40% of all individuals who reported pain. Furthermore, 48% described their pain as mild, 33% as moderate, and 17% as severe. Among those reporting pain in the foot/lower leg, 53% had pain in an additional two or more sites of the body. This differed from individuals who reported pain in other sites except for the foot/lower leg, where only 25% had pain in three or more sites.

**Fig. 1:**
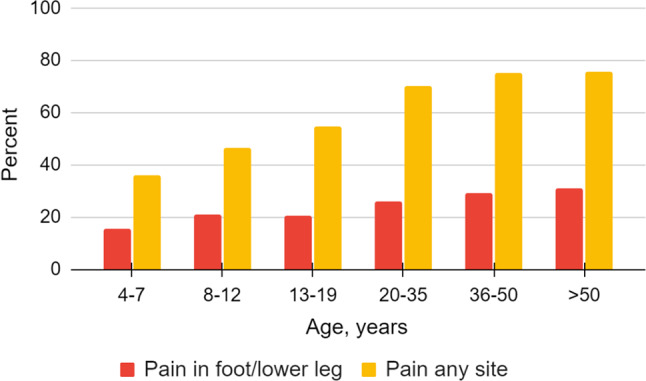
Overall pain and pain in the foot/lower leg related to age in the total study population. A statistically significant increase of pain overall (*p* < 0.001) and pain in the foot/lower leg (*p* < 0.001) with increasing age is shown

**Fig. 2:**
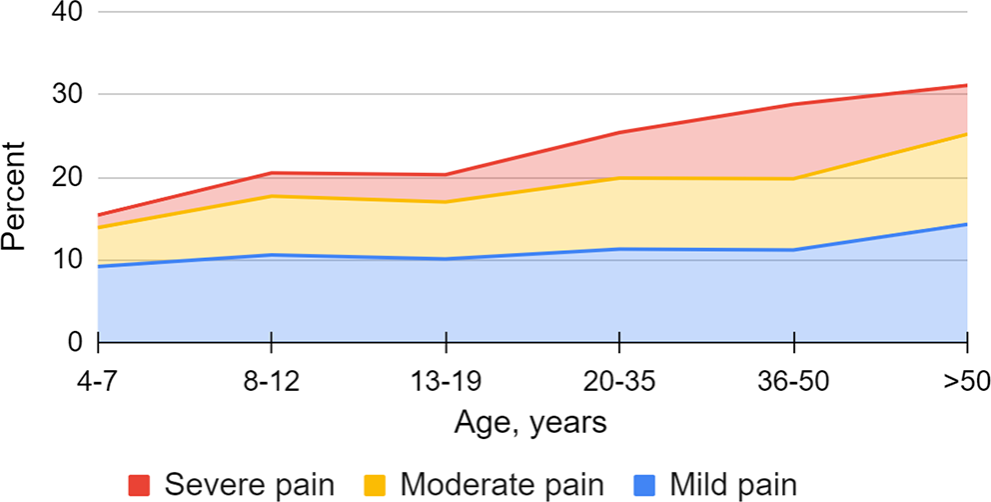
Severity of pain in the foot/lower leg related to age in the total study population (*p* < 0.001)

**Fig. 3:**
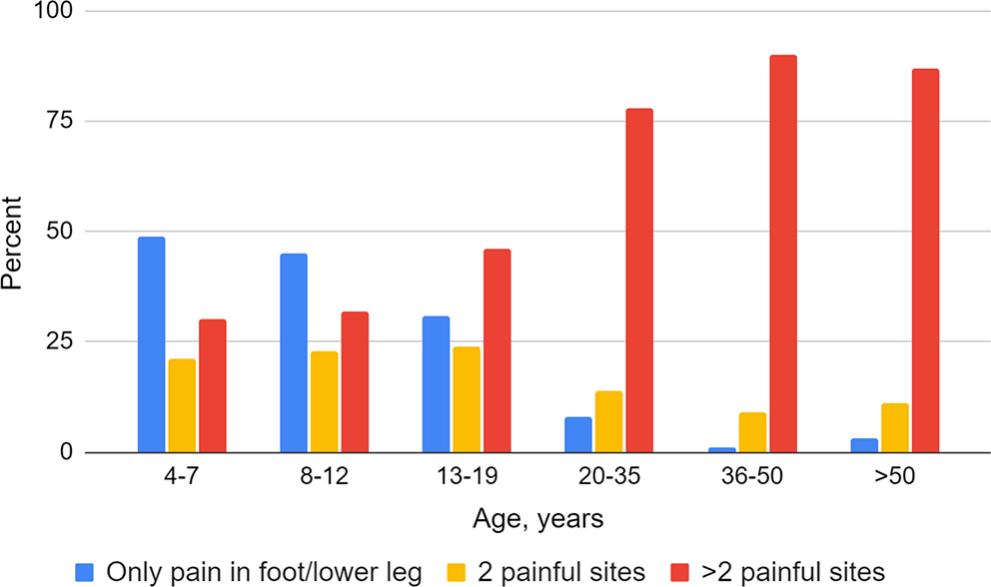
Distribution of the number of painful sites among those reporting pain in the foot/lower leg, related to age

Adults with CP reported pain in the foot/lower leg to a greater extent than those under the age of 18 years (27% vs. 19%, *p* < 0.001), and divided by age categories an increasing trend with higher age was observed (Fig. 1, *p* < 0.001). Adults with pain in the foot/lower leg reported more severe pain (Fig. 2, *p* < 0.001). Regardless of painful site/s, adults more frequently had pain that affected sleep compared to individuals < 18 years (50% vs. 37%, *p* < 0.001), but no statistically significant difference was seen regarding pain that affected ADL. Adults also reported a greater number of painful sites than individuals < 18 years among those reporting pain in the foot/lower leg (Fig. 3, *p* < 0.001) as well as those reporting pain regardless of site (Table 2, *p* < 0.001).

**Table 2:** Pain prevalence in 5122 individuals with cerebral palsy

**n (%)**	**Pain overall, n = 2693**						Pain in foot/lower leg, n = 1077			
	Pain prevalence	Number of painful sites			Pain that affects sleep	Pain that affects ADL	Pain prevalence	Pain intensity		
		1	2	> 2				Mild	Moderate	Severe
**Total population**	2693 (52,6)	1142 (42.4)	529 (19.6)	951 (35.3)	1040 (38.6)	1547 (57.4)	1077 (21.0)	517 (48)	352 (32.6)	182 (16.9)
**Age**										
< 18 years	1485 (45.5)	818 (57.0)	309 (21.5)	309 (21.5)	483 (36.7)	820 (61.7)	608 (18.6)	314 (53.0)	203 (34.3)	75 (12.7)
> 18 years	1208 (70.2)	324 (27.3)	220 (18.5)	642 (54.1)	557 (49.5)	727 (64.6)	469 (27.4)	203 (44.2)	149 (32.5)	107 (23.3)
p-value	< 0.001	< 0.001	< 0.001	0.15	< 0.001	< 0.001				
**Sex**										
Male	1448 (50.5)	642 (45.8)	289 (20.6)	472 (33.6)	522 (40.2)	804 (61.5)	564 (19.7)	274 (50.0)	187 (34.1)	87 (15.9)
Female	1245 (58.9)	500 (41.0)	240 (19.7)	479 (39.3)	518 (45.4)	743 (64.8)	513 (24.3)	243 (48.3)	165 (32.8)	95 (18.9)
p-value	< 0.001	0.009	0.009	0.085	< 0.001	0.44				
**GMFCS**										
I	874 (47.6)	459 (52.9)	157 (18.1)	251 (29.0)	233 (28.8)	473 (58.3)	446 (24.3)	241 (55.8)	134 (31.0)	57 (13.2)
II	512 (56.3)	197 (38.6)	104 (20.4)	209 (41.0)	193 (40.5)	318 (66.1)	255 (28.1)	111 (44.0)	98 (38.9)	43 (17.1)
III	313 (58.8)	102 (33.2)	71 (23.1)	134 (43.6)	128 (44.0)	197 (67.5)	116 (21.9)	48 (43.6)	39 (35.5)	23 (20.9)
IV	432 (54.5)	147 (34.6)	104 (24.5)	174 (40.9)	205 (50.9)	243 (60.4)	135 (17.1)	73 (54.9)	38 (28.6)	22 (16.5)
V	562 (61.6)	237 (46.2)	93 (18.1)	183 (35.7)	281 (61.0)	316 (67.7)	125 (13.8)	44 (35.5)	43 (34.7)	37 (29.8)
p-value	< 0.001	< 0.001			< 0.001	0.001	< 0.001	< 0.001		

Percentages of number of painful sites, pain that affects sleep and pain that affects ADL is calculated from the participants reporting pain, regardless of site. Percentages of pain intensity is calculated from the population reporting pain in the foot/lower leg. Individuals with missing data not included in the calculations. GMFCS: Gross Motor Function Classification System. Statistical significance is calculated using crosstabulation and Pearson chi-square tests

Women/girls reported pain more frequently than men/boys, both overall and in the foot/lower leg (59% and 24% vs. 51% and 20%, *p* < 0.001). Following the Bonferroni adjustment, no statistically significant differences between the sexes were seen on pain intensity, number of painful sites, or in pain that affects ADL/Work or sleep (Table 2).

Individuals who could walk (GMFCS I-III) had fewer reports of pain in general than those who could not walk (52% vs. 58%, *p* < 0.001) but had a higher prevalence of pain in the foot/lower leg (25% vs. 15%, *p* < 0.001) than individuals who could not walk. There was also a statistically significant difference among GMFCS levels on number of painful sites, pain that affects sleep or ADL, as well as pain intensity (*p* < 0.001). The highest number of painful sites was observed in individuals in GMFCS level III, while pain affecting ADL and pain affecting sleep were most prevalent in those in GMFCS level V (Table 2).

Results from the multiple logistic regression are reported in Table 3. The adjusted ORs of pain in the foot/lower leg were statistically significantly higher in females, individuals in GMFCS levels I-II, and in individuals with reduced dorsiflexion of the ankle of ≤ 10 degrees. When age was analyzed as a continuous variable, ORs of pain increased with age. However, when age was categorized into age groups, the adjusted OR of pain showed a statistically significant increase only in individuals > 20 years old. Furthermore, the adjusted ORs of pain in the foot/lower leg were statistically significantly lower in individuals with dyskinetic or ataxic CP and for those who proxy-reported.

**Table 3:** Stepwise multiple logistic regression analysis of pain in foot/lower leg in individuals with cerebral palsy

	Unadjusted	Model 1	Model 2	Model 3	Model 4
	OR (95% CI)	OR (95% CI)	OR (95% CI)	OR (95% CI)	OR (95% CI)
**Sex**					
Male	1	1	1	1	1
Female	1.31 (1.14–1.49)	1.28 (1.12–1.47)	1.31 (1.14–1.50)	1.33 (1.15–1.54)	1.31 (1.13–1.53)
**Age, continuous**					
	1.02 (1.01–1.02)	1.02 (1.01–1.02)	1.02 (1.01–1.02)	1.02 (1.01–1.02)	1.02 (1.01–1.03)
**GMFCS**					
V	1		1	1	1
I	2.00 (1.61–2.49)		1.71 (1.31–2.23)	1.72 (1.29–2.31)	1.84 (1.32–2.57)
II	2.43 (1.92–3.09)		2.02 (1.55–2.62)	2.00 (1.50–2.68)	2.01 (1.46–2.79)
III	1.75 (1.32–2.31)		1.34 (0.99–1.81)	1.32 (0.95–1.84)	1.33 (0.93–1.90)
IV	1.28 (0.99–1.67)		1.15 (0.88–1.52)	1.23 (0.91–1.68)	1.21 (0.87–1.68)
**Subtype**					
Spastic	1		1	1	1
Dyskinetic	0.50 (0.38–0.64)		0.64 (0.49–0.84)	0.67 (0.50–0.90)	0.67 (0.49–0.91)
Ataxic	0.65 (0.45–0.93)		0.59 (0.41–0.86)	0.59 (0.40–0.89)	0.64 (0.43–0.97)
Mixed/non-classifiable	0.69 (0.50–0.96)		0.79 (0.57–1.09)	0.89 (0.63–1.26)	0.95 (0.66–1.36)
**Source of report**					
Report by self	1		1	1	1
Report by proxy	0.54 (0.47–0.62)		0.75 (0.63–0.90)	0.79 (0.65–0.95)	0.78 (0.64–0.94)
**ROM Ankle, degrees**					
> 20	1			1	1
11–20	1.45 (1.18–1.78)			1.19 (0.95–1.48)	1.24 (0.98–1.56)
1–10	1.90 (1.55–2.33)			1.40 (1.12–1.77)	1.43 (1.13–1.83)
≤ 0	2.05 (1.62–2.59)			1.41 (1.08–1.84)	1.46 (1.10–1.93)
**Level of spasticity in ankle plantarflexion**					
Low (MAS 0–1)	1			1	1
High (MAS ≥ 2)	0.97 (0.83–1.14)			0.98 (0.82–1.15)	0.98 (0.82–1.18)
**Botulinium toxin A below-the-knee since last CPUP assessment**					
No	1				1
Yes	1.26 (1.02–1.55)				1.33 (1.05–1.69)
**Spasticity reducing treatment**					
No	1				1
Yes	0.82 (0.68–0.999)				1.13 (0.88–1.45)
**Surgery below-the-knee since last CPUP assessment**					
No	1				1
Yes					1.22 (0.81–1.85)

OR: Odds ratios. GMFCS: Gross Motor Function Classification System. ROM: Range of motion. CPUP: Cerebral palsy follow up program. MAS: Modified Ashworth Scale

No statistically significant difference was seen on prevalence of pain in the foot/lower leg and treatment with botulinum toxin A (BTX-A) below the knee, other spasticity-reducing treatments, or below-the-knee surgery since the last CPUP assessment (Table 4). However, in the multiple logistic regression analysis, the adjusted ORs of having pain in the foot/lower leg were statistically significantly higher in individuals having received BTX-A (Table 3).

**Table 4:** Prevalence of pain in foot/lower leg related to invasive treatments in individuals with cerebral palsy

	Pain in foot/lower leg, n (%)		
	No	Yes	p-value
Surgery below-the-knee since last CPUP* evaluation			0.066
No	3707 (97.3)	1007 (96.3)	
Yes	101 (2.7)	39 (3.7)	
Botulinium toxine below-the-knee since last CPUP* evaluation			0.032
No	3366 (89.4)	898 (87.0)	
Yes	400 (10.6)	134 (13.0)	
**Spasticity reducing treatment**			0.08
No	3098 (83.1)	872 (85.7)	
Oral baclofen	413 (11.1)	86 (8.5)	
Intrathecal baclofen	143 (3.8)	40 (3.9)	
Selective dorsal rhizotomy	53 (1.4)	18 (1.8)	
Selective dorsal rhizotomy + oral baclofen	4 (0.1)	0 (0)	
Unknown if oral or intrathecal baclofen	15 (0.4)	1 (0.1)	

Column percentages reported. CPUP: Cerebral palsy follow-up program

The multinominal regression showed that individuals had higher risk of reporting severe pain than mild or moderate pain in their foot/lower leg with increasing age. Sex was not correlated with pain intensity. Individuals in GMFCS V were more likely to have severe pain in the foot/lower leg compared to individuals in GMFCS I-IV (Table 5).

**Table 5:** Relevant results of multinominal logistic regression analysis on pain intensity of pain in the foot/lower leg

	Severe pain as reference	
	Mild pain	Moderate pain
	RRR (95% CI)	RRR (95%CI)
**Age**	0.98 (0.97–0.99)	0.99 (0.97-0.9996)
**GMFCS**		
I	3.35 (1.82–6.19)	1.84 (0.98–3.47)
II	2.42 (1.29–4.55)	1.94 (1.01–3.69)
III	2.53 (1.19–5.36)	1.98 (0.91–4.29)
IV	3.22 (1.57–6.61)	1.71 (0.79–3.68)
V	1	1

RRR: Relative risk ratio. GMFCS: Gross Motor Function Classification System

## Discussion

Pain in the foot/lower leg affected one in five individuals with CP and was more frequent in adults, in females, in those in GMFCS levels I-II, and in individuals with reduced ROM of the ankle. It was less frequent in individuals with dyskinetic or ataxic CP, as well as in those with proxy-reported pain.

Increased frequency and intensity of pain in the foot/lower leg and a greater number of painful sites were noted with increasing age. Especially in adults, pain in the foot/lower leg seemed to be part of a more complex pain situation with multiple painful sites. Affecting more than 1 in 4 adults, pain in the foot/lower leg might be a contributing factor of the decline in participation, motor function/walking ability, or quality of life that has been described in previous studies on adults with CP [3, 4, 6, 14, 18–22].

The findings that women and girls reported pain more frequently is in line with previous studies [3, 5, 10, 16–18]. However, women and girls do not seem to have more severe pain in their feet/lower legs than men and boys. Parkinson et al. 2013 reported more severe pain in girls, however, that was in general, and not associated with a particular site [10]. Population-based studies have consistently shown that women report pain more often than men and there is increasing evidence that there is a difference between males and females regarding pain sensitivity and analgesic response. However, pain intensity is more often equal between the sexes. The underlying causes for this are still unclear but a combination of biological, psychological, and sociocultural factors is likely to contribute [29, 30]. 

The higher prevalence of pain in the foot/lower leg among those who ambulate differ from previous studies on pain, where pain generally was more common in higher GMFCS levels [3, 6, 14, 18]. However, individuals in GMFCS level V were more likely to have more severe pain in the foot/lower leg.

Of the risk factors for pain in the foot/lower leg, sex, age, and GMFCS-level are largely non-modifiable. However, a factor that is modifiable is reduced ROM of the ankle. Nevertheless, due to the cross-sectional design of this study, it was not possible to determine if the decreased ROM caused pain, or if the ROM was decreased because of pain.

Overall, that individuals who put weight on their feet are at higher risk of pain in the foot/lower leg is clinically plausible. Although we cannot deduce the exact cause of pain, one hypothesis is that putting weight on a joint at the extreme end of its ROM is a strain. Moreover, a deformed foot does not tolerate as much strain or weight as a non-deformed foot.

Dyskinetic CP has previously been associated with a higher prevalence of pain – regardless of site – [14, 18], however, we observed lower odds of pain in the foot/lower leg in individuals with dyskinetic and ataxic CP compared to individuals with spastic CP (Table 3), even after adjusting for GMFCS level and ROM. This was somewhat unexpected, and we are not able to completely explain this finding. It is possible, however, that the muscle tone is more variable in those with dyskinetic and ataxic CP, which might be associated with less pain than the more persistent increased muscle tone associated with spastic CP.

In this study, we have not looked specifically on presence of a coronal plane deformity of the foot. Like equinus, valgus and varus deformities are caused by the combination of muscle imbalance, impaired motor control, abnormal tone, and gravity and range from mild and flexible to severe and rigid [31, 32]. Type of deformity varies with the degree of motor involvement and CP subtype. We do not know if the type of deformity affects pain prevalence, but it is a reasonable thought that should be further researched.

We found that individuals with pain in their foot/lower leg were more likely to have received local spasticity-reducing injections with BTX-A since their last CPUP assessment. No statistical difference was seen regarding other spasticity reducing treatments, such as oral or intrathecal baclofen, or selective dorsal rhizotomy. BTX-A is injected to reduce muscle tone of the treated muscle, and one indication for this treatment in CP is painful spasticity. The increased odds of having pain in the foot/lower leg in those who have been treated with BTX-A may be attributed to pain being an indication for BTX-A treatment. Nevertheless, we do not know exactly where the injection was given or when in the treatment cycle. Further studies with a longitudinal approach regarding the effect of interventions such as BTX-A on pain are needed.

Although no clear association has been found, it is possible that other treatments, such as orthotic devices, might play a part in the painful foot/lower leg. Ankle-foot orthoses (AFO) are commonly used in children with CP, and while there is limited evidence that AFOs might have a beneficial effect on ROM not much is known regarding positive or negative effects on pain [33–35]. However, we did not have enough data on use of orthotic devices on the lower leg/foot to include it in our analyses.

### Study limitations

There are several limitations in this study. The coverage rate of adults with CP is less complete than for children: 15% leave CPUP after turning 18 years. A majority of those who decline to continue in CPUP are in GMFCS level I, and as such are at a higher risk of developing pain in their feet/lower legs. Although this is a potential inclusion bias that might affect the pain prevalence, GMFCS is adjusted for in the regression analyses. An additional limitation is that 43% of the participants did not self-report. Previous studies have indicated that proxies tend to report pain to a lesser degree in adults, and tend to report lower pain intensity scores [12, 16, 17, 36]. It is plausible to assume that the interpretation of pain is even harder for proxies in individuals with multiple painful sites.

In this study, we focused on the main effects of our exposure variables and did not assess potential interactions between our exposure variables in the multiple logistic regression analysis which could provide further insights.

While there are clear advantages with register-based research in regards to, for example, generalizability, one drawback tends to be the lack of details in the reported data. In other words, in smaller studies it might be more feasible to collect data at a more detailed level. Hence, more research is required to investigate in more detail the relationship between, for instance, subtype of CP, spasticity, foot deformities, and pain in the foot/lower leg.

Overall, to experience pain in the foot/lower leg seems to be part of a more complex picture; more than half of those with pain in the foot/lower leg reported pain in three or more sites. In comparison, more than half of the individuals who had pain somewhere else, but not in the foot/lower leg, reported only one painful site. In this context, this is a limitation making us unable to tease out what effect only pain in the foot/lower legs has on ADL/Work or sleep, since the data do not specify how each painful site affects ADL/Work or sleep.

## Conclusions

Pain in the foot/lower leg is a common problem in individuals with CP. Adults, women, and individuals who are ambulatory were at higher risk of having pain in the foot/lower leg. Pain in the foot/lower leg was associated with decreased ROM of the ankle. Moreover, pain in the foot/lower leg is often combined with pain in other sites. Overall, our findings support that just like CP is a heterogeneous condition, so is CP-related pain. To that end, given that risk factors for pain seem to differ between different parts of the body, both clinicians and researchers should approach each painful body site separately. Given that one in two individuals with CP report pain, and one in five report pain in the foot/lower leg, we must strive for early identification, evaluation, and treatment of these individuals to maintain function and prevent deterioration of mobility and quality of life.

## Data Availability

Data used in this study are owned by Skane Regional Council (Region Skane, 29,189 Kristianstad, Sweden). The datasets used and/or analyzed during the current study are available from the corresponding author on reasonable request and with permission of the Skane Regional Council.

## Abbreviations

CP: Cerebral Palsy
CPUP: Cerebral palsy follow-up program
OR: Odds ratio
CI: Confidence interval
ADL: Activities of daily living
GMFCS: Gross motor function classification system
ROM: Range of motion
MAS: Modified Ashworth scale
IQR: Interquartile range
VIF: Variance Inflation factor
RRR: Relative risk ratios
BTX-A: Botulinium toxin A
AFO: Ankle-foot orthosis
